# A Review of the Effect of Trace Metals on Freshwater Cyanobacterial Growth and Toxin Production

**DOI:** 10.3390/toxins11110643

**Published:** 2019-11-05

**Authors:** Jordan A. Facey, Simon C. Apte, Simon M. Mitrovic

**Affiliations:** 1 Freshwater and Estuarine Research Group, School of Life Sciences, Faculty of Science, University of Technology Sydney, Ultimo 2007, Australia; 2 CSIRO Land and Water, Lucas Heights 2234, Australia

**Keywords:** cyanobacteria, freshwater, trace metals, blooms, toxin production, growth limitation

## Abstract

Cyanobacterial blooms are becoming more common in freshwater systems, causing ecological degradation and human health risks through exposure to cyanotoxins. The role of phosphorus and nitrogen in cyanobacterial bloom formation is well documented and these are regularly the focus of management plans. There is also strong evidence that trace metals are required for a wide range of cellular processes, however their importance as a limiting factor of cyanobacterial growth in ecological systems is unclear. Furthermore, some studies have suggested a direct link between cyanotoxin production and some trace metals. This review synthesises current knowledge on the following: (1) the biochemical role of trace metals (particularly iron, cobalt, copper, manganese, molybdenum and zinc), (2) the growth limitation of cyanobacteria by trace metals, (3) the trace metal regulation of the phytoplankton community structure and (4) the role of trace metals in cyanotoxin production. Iron dominated the literature and regularly influenced bloom formation, with 15 of 18 studies indicating limitation or colimitation of cyanobacterial growth. A range of other trace metals were found to have a demonstrated capacity to limit cyanobacterial growth, and these metals require further study. The effect of trace metals on cyanotoxin production is equivocal and highly variable. Better understanding the role of trace metals in cyanobacterial growth and bloom formation is an essential component of freshwater management and a direction for future research.

## 1. Introduction to Cyanobacteria in Freshwater Systems

Throughout the world, there is an increasing demand for freshwater utilised for irrigation, industry, recreation and direct consumption [1]. Satisfying both ecological and anthropogenic water requirements is challenging and may prove more difficult in the context of climate change and a growing human population [2]. The proliferation of toxin-producing cyanobacteria (blue-green algae) poses a significant threat to the integrity of freshwaters and their functions [3]. Under favourable environmental conditions, cyanobacteria can dominate the phytoplankton community and form high cell density blooms and scums [4]. Thick surface blooms cause a reduction of water clarity, decreasing oxygen production in the bottom layers of the water column and suppressing macrophyte growth, which can negatively affect invertebrate and fish habitats [5]. Bacterial decomposition of senescent blooms can also cause anoxic conditions, or blackwater events, often leading to fish kills [6,7].

Some bloom-forming cyanobacteria produce toxic secondary metabolites called cyanotoxins [6,8]. Cyanotoxin-containing blooms occur throughout the world and are responsible for sporadic episodes of animal illness and death, as well as human poisonings from municipal and recreational water supplies [9,10,11]. Cyanotoxins are highly variable in terms of their molecular structure, production triggers and modes of toxicity [4,11,12]. Effects range from skin irritation to cancer or even fatalities [3,13]. For example, epidemiological research in Central Serbia has established a link between cyanobacteria blooms in drinking water reservoirs and increased incidence of liver cancer in regions consuming this water source [14]. In Australia, Pilotto et al. [15] observed that exposure to cyanobacteria during recreational water-related activities was associated with gastrointestinal disturbances, flu-like symptoms, skin rashes, mouth ulcers, fevers and eye or ear irritations up to seven days after exposure. Symptom occurrence was correlated with increased duration of water contact and higher cyanobacterial cell counts.

There is an increasing frequency, severity and geographic extent of cyanobacteria blooms, which can be attributed to the dominance of cyanobacteria in anthropogenically modified aquatic ecosystems [6,16,17]. Increased temperature, nutrient pollution and low-velocity flow regimes promote the development of dense, toxic, cyanobacteria blooms [18,19,20,21]. This trend is likely to continue as cyanobacteria are expected to flourish under the environmental conditions predicted for global climate change [22,23,24] and toxic cyanobacterial taxa are comprising an increasing proportion of the phytoplankton community under bloom conditions [19,22].

## 2. Nutrient Limitation

The availability of key nutrients can greatly influence the phytoplankton community composition in surface waters [25]. Low levels of the macronutrients phosphorus (P) and nitrogen (N) are frequently the limiting factor of cyanobacterial growth in freshwater ecosystems [26,27,28], and therefore, N and P inputs can stimulate cyanobacterial bloom formation [25,29]. Generally, low N and P concentrations promote a highly diverse, low biomass phytoplankton community, often associated with good water quality [25]. Conversely, high N and P concentrations, or eutrophication, regularly promote the formation of dense cyanobacterial surface blooms and subsequent deterioration of water quality [25,26,27].

Since the 1970s, phosphorus reduction has been the most widely adopted solution to eutrophication [30]. On the other hand, more recently, nitrogen reduction or dual nutrient control is being widely implemented [26,27,31]. However, there are instances where high P and N concentrations and seemingly favourable conditions do not produce blooms, suggesting there are unknown bloom triggers [32]. While the link between nitrogen and phosphorus and cyanobacteria growth is well established, there is growing evidence that phytoplankton growth (including toxic bloom-forming cyanobacteria) can also be limited by micronutrient trace metals, alone or in combination with macronutrients [33,34,35,36,37]. This may help explain the occurrence of blooms in mesotrophic systems [35]. Currently, the role of trace metal micronutrients in cyanobacterial bloom formation is often overlooked as trace metals are rarely considered in eutrophication management strategies. Identifying sources of trace metals and how they impact phytoplankton communities may be important in understanding toxic cyanobacterial bloom dynamics.

## 3. Sources of Nutrients

As with other nutrients, trace metal concentrations in aquatic systems are highly variable in space and time [38]. Inflows to a waterbody, such as floods and heavy rain, can mobilise allochthonous (catchment) sources of the macronutrients nitrogen and phosphorus [39] and metals [38,40]. These events can have significant effects on primary productivity and can alter phytoplankton community structure [41]. Changing land use practices and anthropogenic point sources of pollution (such as stormwater or irrigation drains) can also elevate macronutrient and trace metal concentrations in waters [42,43,44,45].

Despite recent advances in biological phosphorus removal, wastewater can be a significant source of macronutrients in aquatic ecosystems [45,46]. Wastewater treatment plants can also be ineffective at removing all trace metals and can act as a source of these potential micronutrients [47,48]. The influence of wastewater discharge on phytoplankton was examined by Luoma [48], who estimated that ≈60% of the total input of Cd, Ni and Zn from wastewater treatment plants is cycled through the phytoplankton community in a bay subject to regular blooms. It is likely that bloom dynamics can be influenced by wastewater treatment discharge containing both trace metals and macronutrients.

Sediments act as both a source and a sink for nutrients including trace metals in aquatic ecosystems and play a significant role in determining nutrient availability [36,49]. Thermal stratification of the water column often causes hypoxia below the thermocline, stimulating the release of nutrients such as phosphorus, nitrogen and iron from anoxic sediments [49,50,51]. Thermally stratified conditions also favour the proliferation of cyanobacteria whose buoyancy regulation may allow vertical migration to access nutrients at the sediment/water interface [22,36,52,53]. Additionally, when the waterbody undergoes a mixing event, the nutrient rich hypolimnial water is transported to the surface via upwelling, thereby increasing nutrient availability to cyanobacteria [50]. For example, a cyanobacterial bloom in the Fitzroy impoundment near Rockhampton, Australia, was at least partially attributed to upwelling of nutrient-rich, anoxic, hypolimnetic waters into the surface layer. This large nutrient source supported a bloom of mixed small cyanobacteria species that persisted for over three months [53].

## 4. Colimitation and Optimal Nutrient Ratios

The traditional view of nutrient limitation is derived from Liebig’s law of the minimum, stating that productivity is limited by the nutrient that is least available relative to the organism’s overall nutritional requirement [54,55]. This implies that only a single resource is ever limiting at one time; for example, Schindler et al. [56] suggested that reducing phosphorus input alone is effective at reducing harmful algal blooms.

However, two simultaneously added nutrients can sometimes stimulate a larger response than their individual additions, suggesting colimitation by both nutrients and the need for dual nutrient management [27,28,55,57]. Harpole et al. [55] distinguishes between simultaneous and independent colimitation. When the addition of two nutrients or resources in combination elicits a response, but there is no response to their individual additions, this is classified as simultaneous colimitation. On the other hand, independent colimitation refers to a greater response to resources added in combination than the response to individual additions.

Beyond total nutrient supplies, the ratio of two or more resources can also affect nutrient limitation [58]. For example, the Redfield ratio describes the stoichiometry of nutrients in the cytoplasm of marine phytoplankton that allows optimal growth and metabolism [59]. When optimal nutrient ratios are not met (e.g., one nutrient is supplied at a suboptimal concentration relative to another nutrient), growth and productivity are limited. While the Redfield ratio was originally based on the concentration of nitrate and phosphorus in seawater, this relationship has been extended to include some trace metals such as cobalt [60] and zinc [61]. However, these relationships have not been thoroughly investigated in freshwaters.

## 5. Importance of Trace Metals

The essentiality of trace metals to living organisms is well known. Up to a third of all microbial proteins contain a metal cofactor [62]. Calcium (Ca), copper (Cu), iron (Fe), potassium (K), magnesium (Mg), manganese (Mn), molybdenum (Mo), sodium (Na) and zinc (Zn) are essential to the functioning of the vast majority of organisms. Others, such as barium (Ba), cobalt (Co), nickel (Ni), strontium (Sr) and vanadium (V), are required by just some species [4]. Cyanobacteria have relatively high metal requirements for optimal growth compared to other bacteria largely due to metal cofactors in the oxygenic photosynthetic electron transfer apparatus, such as cytochromes, plastocyanin and chlorophyll rings [63]. An adequate supply of trace metals is required to maintain optimal growth, particularly as these higher metal requirements make cyanobacteria more prone to trace nutrient limitation [4,64]. 

Often metal limitation can occur even when total metal supply is high [4,65]. Many metals cycle between different oxidation states, which have different solubilities and form specific complexes which may not be bioavailable [65]. The speciation of the metal in solution (i.e., its physicochemical form) controls its bioavailability, and therefore its status as a limiting nutrient [66]. For example, the highly bioavailable ferrous iron (Fe^2+^) is very soluble in anoxic waters but is rapidly oxidised to the poorly soluble and non-bioavailable ferric iron (Fe^3+^) in circumneutral oxygenated waters [4,36,67,68].

A growing body of literature demonstrates the impact of trace metals (alone or in combination with macronutrients) on phytoplankton growth [34,69,70,71,72]. For example, Downs et al. [35] observed that the addition of Cu, Mo or Co during a cyanobacterial bloom in a eutrophic lake stimulated primary productivity by up to 40%, indicating a large contribution of micronutrients to eutrophication. Furthermore, North et al. [34] observed that phytoplankton in offshore, thermally stratified regions of Lake Erie were at times colimited by iron, phosphorus and nitrogen. Enrichment with a combination of Fe, P and N stimulated a greater increase in phytoplankton biomass than the nutrients added individually, or compared to a P+N treatment. These findings are reflected in similar experiments by Twiss et al. [33] in Lake Erie and Vrede and Tranvik [69] in several oligotrophic lakes in Sweden. Moreover, rare earth elements (REE), which are utilised in various modern products and technologies, can be released into waters in relatively large quantities [73]. These elements may also be important factors in regulating cyanobacterial bloom formation. For example, a recent study by Shen et al. [74] suggested that lanthanum may impact the growth and microcystin production of *Microcystis aeruginosa*.

Metal requirements within the phytoplankton community, and even within phyla, are highly specific. Therefore, metal availability is a strong determinant of phytoplankton community composition [35,65,75,76]. Community colimitation can occur when one segment of the phytoplankton community is stimulated by a particular nutrient and other segments are not [77]. For example, de Wever et al. [64] noted that iron additions stimulated growth of cyanobacteria in Lake Tanganyika, East Africa, but did not stimulate diatoms or chlorophytes, suggesting cyanobacteria were more sensitive to a decrease in Fe availability compared to other phytoplankton. On the other hand, Zhang et al. [37] showed limitation or colimitation of cyanobacteria by Co, Cu and Fe, and a shift in the phytoplankton community during a nutrient amendment mesocosm at Lake Taihu, China. 

## 6. Iron

**Figure 1:**
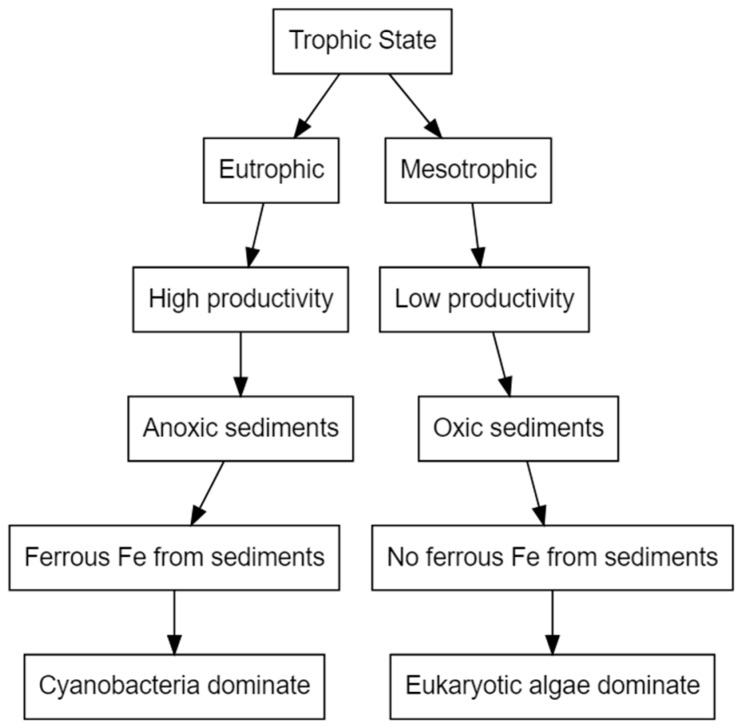
Simplified diagram illustrating how Fe and macronutrient dynamics may interact to alter phytoplankton community dynamics in lakes, reproduced from [36]. 2014, John Wiley & Sons Ltd.

Of all trace metals, iron is required in the greatest quantity and most often limits algal growth (Table 1) [65]. Iron is particularly important to cyanobacteria due to its direct involvement in chlorophyll a synthesis, respiration, nitrogen fixation and photosynthesis [68,78]. It catalyses many biochemical reactions as a cofactor of enzymes, detoxifies reactive oxygen species and has a role in electron transport [34,78,79]. Severe iron limitation reduces the capacity of phycobilisomes to utilise excess light energy, and leads to the formation of reactive oxygen species and subsequently to oxidative stress [68]. Iron availability is a determinant of the dominance of cyanobacteria over eukaryotic species due to the high iron requirements of cyanobacteria, particularly N_2_-fixing species [36,80,81]. Figure 1 illustrates a simplified mechanism of how the trophic state of a lake system can influence iron availability and, subsequently, phytoplankton community structure.

The chemical form of iron strongly influences its bioavailability, toxicity, environmental fate and transport [54,82,83]. Despite being one of the most abundant elements, iron deficiency is a regular source of stress in biological systems [4,82]. As Fe^2+^ is rapidly oxidised in circumneutral water, iron limitation can readily occur in systems lacking internal Fe^2+^ loading from anoxic sediments [4,36,67,68]. However, Fe^2+^ can also be sourced from extracellular photoreduction of Fe^3+^ complexed to dissolved organic matter (DOM) [36]. Some cyanobacteria can overcome the low bioavailability of particulate Fe^3+^ by producing siderophores—low molecular weight metallophores which chelate and solubilise Fe^3+^, but also to Zn, V, Mo, Mn, Co, Ni and Cu [84,85]. Siderophores can enhance the bioavailability of metals and aid in their acquisition from the surrounding environment [85]. The ability of some cyanobacterial genera to produce siderophores may represent a response to a higher degree of sensitivity to low metal availability, particularly Fe, relative to other phytoplankton groups [64].

## 7. Zinc

Zinc is an essential element to cyanobacteria and plays a role in numerous physiological processes, yet, similar to other trace metals, it is also toxic at high concentrations [4,35]. Zinc maintains protein structure and aids in CO_2_ transfer and fixation in the enzyme carbonic anhydrase and in alkaline phosphatase, an enzyme that acquires phosphorus from organic phosphate esters [4,65]. It is also a component of zinc finger proteins, which are needed for DNA transcription [65]. At high concentrations, such as near sewage or industrial effluent outlets, zinc can inhibit phytoplankton productivity and species richness by outcompeting other essential trace metals at binding sites [35,67,76,86].

Zinc availability is generally controlled by the concentration of free metal ions or dissolved inorganic species in the environment, as organic complexes are not readily available to phytoplankton [65]. Due to the involvement of zinc in CO_2_ transfer, cellular requirements increase under CO_2_ limited conditions. During blooms where CO_2_ is largely consumed, cells may become colimited by zinc and CO_2_ [65]. Similarly, given the importance of zinc in phosphate acquisition, algal growth may be colimited by zinc and phosphate in environments where both nutrients occur at low concentrations [65].

## 8. Copper

Copper is essential to cyanobacteria as a micronutrient. It is a component of cytochrome oxidase and plastocyanin in the electron-transport chain, converting light to chemical energy [65,67]. It also facilitates H_2_O dehydrogenation and O_2_ evolution in the thylakoid lumen [79,87]. As with other metals, copper exists in many forms, such as free ions, inorganic complexes and chelates with organic ligands such as fulvic and humic acids [65]. Free ionic copper is the most bioavailable to phytoplankton [88].

At high concentrations, copper can be highly toxic to cyanobacteria, causing a hyperoxidative state, chlorosis and inhibiting growth [89].Because of its toxic effects, copper has been commonly utilised as an algaecide to treat blooms in lakes and reservoirs [90]. Elevated copper concentrations in surface waters are often linked to human activity, due to its presence in antifouling paint, wood preservatives or from municipal waste [67,89]. Lehman et al. [88] found that copper additions as small as 1 µgL^−1^ suppressed phytoplankton growth in the Great Lakes, indicating that in some instances ambient concentrations may already be at the threshold for toxicity to algae and other taxa. In contrast, Zhang et al. [37] observed that the addition of 20 μg/L Cu had a stimulatory effect on algal growth, including *Microcystis aeruginosa*, in the hypereutrophic Lake Taihu.

## 9. Molybdenum

Molybdenum is required for the assimilation of inorganic nitrogen and is therefore particularly important to heterocystous cyanobacteria [71,91]. It is a cofactor in the N_2_-fixing enzyme nitrogenase, among others [29,91,92]. The absence of molybdenum from growth media regularly causes N-limitation in heterocystous cyanobacteria [93] and as such, molybdenum facilitates the introduction of nitrogen into the food web and low molybdenum concentrations can cause colimitation of phytoplankton growth alongside nitrogen [71].

Molybdenum generally occurs as the oxyanion MoO_4_^2−^ in natural waters, in concentrations typically of less than 20 nmol/L (less than ≈2 μg/L) in freshwater environments [94]. These low molybdenum concentrations are often insufficient for optimal nitrogen fixation by heterocystous cyanobacteria [91,95]. Contributing to this deficiency, competitive inhibition of transport proteins by sulfate further limits molybdenum availability to N_2_-fixing cyanobacteria [91,94]. The interactions between molybdenum and sulfate may cause a switch in the nutrient requirements of phytoplankton along a salinity gradient. Howarth and Cole [96] outline a general trend of phosphorus limitation in inland freshwater and nitrogen limitation in sulfate-rich coastal waterways due to inhibited molybdenum assimilation. However, Paerl and Fulton [29] suggest that some cyanobacteria possess nitrogenases that do not require molybdenum and they would therefore have a way of circumventing low molybdenum availability.

## 10. Cobalt

A number of studies have assessed the cobalt requirements of marine cyanobacteria and concluded that Co can act as a determinant of marine cyanobacteria distribution and productivity [62]. However, micronutrient requirements often differ between marine and freshwater cyanobacteria [97]. Downs et al. [35] noted a stimulation of primary productivity upon addition of cobalt during a bloom of the freshwater heterocystous cyanobacteria *Anabaena flos-aquae*. Yet, the importance and role of cobalt in freshwater cyanobacterial species is severely understudied.

Cobalt is often associated with vitamin B_12_, a diverse group of corrinoids involved in the transfer of methyl groups and rearrangement reactions in cellular metabolism [62,92,98,99]. B_12_ is required by the majority of microalgae for growth, but it can only be synthesized de novo by certain prokaryotes, including most cyanobacteria [99]. However, recent work by Helliwell et al. [99] demonstrates that pseudocobalamin, which is relatively non-bioavailable, is the dominant form produced by cyanobacteria, suggesting a complex B_12_ cycle in aquatic systems. Rodriguez and Ho [98] conducted batch cultures of *Trichodesmium* with varying concentrations of Co and vitamin B_12_. Low cobalt concentrations appeared to limit *Trichodesmium* growth. Upon addition of vitamin B_12_, growth was elevated. These results support cobalt requirements for vitamin B_12_ synthesis in some cyanobacteria. Interestingly, vitamin B_12_ deficiency appears to promote nitrogen fixation of marine cyanobacteria, perhaps because vitamin B_12_ is a nitrogen-rich molecule [92,98]. 

Cobalt can substitute for other micronutrients, such as zinc and cadmium. For example, the marine diatom *Contricribra* (*Thalassiosira*) *weissflogii* can utilise Co in place of Zn in the enzyme carbonic anhydrase [97]. When both micronutrients are available, Zn is favoured [97,100]. However, some marine cyanobacteria (e.g., *Prochlorococcus*, *Trichodesmium* and *Synechococcus*) appear to have an absolute cobalt requirement [98,101,102]. For example, Rodriguez and Ho [98] showed that *Trichodesmium* has an absolute cobalt requirement that cannot be alleviated by the addition of zinc. Saito et al. [102] observed a similar phenomenon in the cyanobacterium *Prochlorococcus*.

Ji and Sherrell [103] observed that *Microcystis* sp. subjected to phosphorus limitation exhibited an increase in both cellular Co and alkaline phosphatase (APase) activity. When cyanobacteria are subjected to extended phosphorus deficiency, extracellular APase is excreted to catalyse the hydrolysis of dissolved organic phosphorus when the preferred inorganic phosphorus is limited [103,104]. The dominant phosphatase in *Microcystis* may require cobalt, as reported for other prokaryotes, and may be accumulated upon phosphate deficiency due to the upregulated activity of APase [103].

## 11. Manganese

Manganese is one of the most abundant transition metals on earth and is required by all known organisms [105]. Manganese exists in various chemical forms, predominantly as the highly soluble and bioavailable Mn(II) ion [105] and also as Mn(III) and Mn(IV), which are present mainly in particulate forms which are insoluble and non-bioavailable [66]. Similar to iron, manganese is essential for photosynthesis due to its role in the thylakoids, where four manganese atoms are required by every water-splitting oxygen-evolving complex in Photosystem II [65,67,79]. Despite the importance of manganese, it is generally not considered to limit phytoplankton growth or primary productivity in aquatic ecosystems due to its high abundance [105]. However, Salomon and Keren [105] indicated that even small changes in the natural ambient concentrations of manganese can impose changes in photosynthetic activity of the freshwater cyanobacterium *Synechocystis* sp. Kraemer et al. [84] suggest that siderophores may play a role in manganese biochemistry, primarily by forming Mn(III)-siderophore complexes, thereby increasing manganese availability to cyanobacteria. 

## 12. Cyanotoxin Production

The increasing prevalence of toxic cyanobacterial blooms has led many researchers to investigate the causes and stimulants of toxin production [86,106,107,108,109,110,111,112]. The complex structure and high energetic cost of cyanotoxin production is only justified if they confer some benefit to the producing organism [109]. The benefits of cyanotoxins have been demonstrated in a number of studies, for example, competition experiments conducted by Briand et al. [113] showed that microcystin-producing strains of *Planktothrix agardhii* were more successful than non-microcystin-producing strains under limiting temperature, light and nitrate conditions. On the other hand, under favourable conditions the non-toxic strain was more successful, suggesting that the energetic cost of producing microcystin outweighed the benefit. Further, a genetic study by Zilliges et al. [114] noted increased transcription of *mcy* mRNA when *Microcystis* was exposed to high light, iron limitation and other oxidative stress conditions. They suggested microcystin-producing strains of *Microcystis* have an advantage over non-toxic strains under oxidative stress conditions due to a protein-modulating role of microcystin.

However, the precise role of cyanotoxins remains highly contentious. Given the deleterious effect of cyanotoxins on a multitude of organisms, it is perhaps logical to conclude that cyanotoxins are produced as a grazing deterrent or to reduce competition [11]. As observed by Rohrlack et al. [115], cyanotoxins can act as an antipredator defence mechanism as a toxin-producing strain of *Microcystis* was lethal to *Daphnia*, whereas a mutant deficient of the microcystin biosynthesis genes (*mcy*) did not have lethal effects. However, defence against grazers is unlikely to be the primary function of cyanotoxins due to the early evolution of the genes responsible for their synthesis, prior to the evolution of metazoans and the subsequent grazing pressure [111,116]. The toxic effects of microcystin may have aided in the retention of microcystin biosynthesis genes or may be a more recently evolved secondary function.

Cyanotoxin production, particularly microcystin, has been widely studied as a function of various physiochemical properties in an attempt to understand their possible functions, for example, macronutrients [112,117], radiation, pH and temperature [118,119] and some trace metals [86,106,108,109]. Often toxin production is simply correlated with cell division and growth, suggesting that there is no direct effect on the metabolic pathway [108,117,118,120], whereas in others, a relationship appears [86,112,121]. Neilan et al. [119] reasoned that while there is a strong correlation between toxin production and growth rate, a more complex relationship with some physiochemical conditions exists.

## 13. Trace Metals and Cyanotoxins

Some cyanotoxins form complexes with metal ions (Fe^2+^, Zn^2+^, Cu^2+^, Mg^2+^), and consequently there have been suggestions that this points to their role in nature as trace metal-complexing ligands [122,123]. If trace metal availability influences the rate of cyanotoxin production, metals may be an important regulator of the toxicity of blooms [68]. Birch and Bachofen [124] state that complexing ligands produced by microorganisms are usually part of a transformative, detoxifying process. Cyanotoxins may therefore be produced in response to high trace metal concentrations as a means of detoxification [125]. Huang et al. [126] observed the effects of toxic levels of cadmium on *Microcystis aeruginosa* and found no evidence that microcystin can affect metal toxicity by regulating metal accumulation or by directly assisting in the detoxification.

Alternatively, metals could be complexed by cyanotoxins as a means of acquisition or storage. Lukac and Aegerter [109] found that trace metal concentration influenced the production of microcystin in *Microcystis aeruginosa*. Severe iron and zinc limitation increased toxin production, indicating that microcystin may function as an intracellular chelator aiding in trace metal accumulation. This hypothesis is supported by Yeung et al. [107], who also observed higher intra- and extracellular microcystin quotas in iron-limited *Microcystis* cultures. Furthermore, Sevilla et al. [82] found that iron starvation increased transcription of the *mcyD* gene involved in microcystin synthesis, and Polyak et al. [86] noted that concentrations of 25–100 μg/L Zn^2+^ increased intracellular microcystin concentration. However, a number of studies have found that trace metals have no effect on cyanotoxin production. For example, Harland et al. [72] studied anatoxin-a production by *Phormidium autumnale* and found no relationship with iron or copper concentrations. Similarly, Gouvêa et al. [108] suggests that toxin production paralleled specific growth rate and biomass rather than being directly influenced by metals. 

Chelators often enhance the availability of metals to phytoplankton by maintaining them in a soluble, diffusible form and preventing precipitation or adsorption onto particle surfaces [69]. The acquisition hypothesis implies that cyanotoxins function similarly to siderophores, molecules that are actively transported across the cell membrane to form strong extracellular complexes with ferric iron and increase iron bioavailability via a reduction reaction to form ferrous iron [29,68,125,127]. Klein et al. [128] showed that Fe^3+^ forms weaker complexes with microcystin-LR than is typical of other siderophores, and proposed that microcystin is more likely to regulate iron via intracellular processes or by acting as a shuttle across the cell membrane. Another feature of siderophores which is not observed in microcystin is the lack of active extracellular translocation [108,114]. Despite the identification of a putative microcystin ABC transporter, the majority of microcystin (>90%) is released only upon cell lysis [129]. Moreover, Fujii et al. [130] compared a microcystin-producing strain of *Microcystis* and an *mcyH* deficient mutant and found that microcystin did not facilitate iron uptake in the microcystin-producing strain. These observations point towards a primary intracellular role for microcystin, perhaps by acting as transporters, increasing membrane permeability, forming complexes on the cell surface or increasing phagocytic ability of algal cells [123,131]. 

## 14. Knowledge Gaps

Given the ability of cyanobacteria to form blooms and produce toxins, they are of particular importance to catchment managers. While a large number of studies demonstrate trace metal limitation of primary productivity in freshwater (see review by Downs et al. [35] and more recent studies such as Harpole et al. [55] and Corman et al. [141]), relatively few studies assess the effect specifically on freshwater cyanobacteria. Cyanobacteria have particular trace metal requirements and metal uptake strategies [64,81]. Therefore, metals may stimulate growth in the cyanobacterial community but decrease overall phytoplankton productivity. It is important to differentiate between the cyanobacterial response and the response of the overall community. Furthermore, understanding how cyanobacteria compete with other phytoplankton groups under different trace metal and macronutrient regimes has received little attention, although Molot et al. [36,81] and Sorichetti et al. [139] do provide conceptual models for iron-mediated bloom formation and community dynamics.

Of the 27 studies presented in Table 1, 12 focus on metal interactions with *Microcystis* spp. This may be as *Microcystis* is the most common bloom-forming genera [142,143,144,145,146] and is therefore central to many catchment management plans [27,147] and axenic cultures are readily available. However, cyanobacteria are a diverse group, with upwards of 150 genera [148], and literature skewed towards *Microcystis* does not reflect the overall cyanobacterial community. Similarly, microcystin dominates the literature in studies of environmental regulation of cyanotoxin synthesis [142]. However, there is a high degree of structural variation in bioactive, toxic compounds released by cyanobacteria [149], which suggests that the factors stimulating cyanotoxin production and their biological role may be unique to each compound. This body of knowledge must be expanded by the addition of other cyanobacterial species and cyanotoxins to better understand the role of metals in the growth of cyanobacteria and provide insight into species-specific responses.

Iron is by far the most commonly examined trace metal and most frequently observed metal to effect cyanobacterial growth, as is evident in Table 1. Of the 18 studies which examine iron’s effect on cyanobacterial growth, 15 observed limitation or colimitation. While all trace metals examined in this review have a demonstrated capacity to limit cyanobacterial growth to some degree, they have not received the same attention. For example, cobalt’s effect on freshwater cyanobacterial growth has only been examined in five studies, of which one showed limitation. Similarly, the influence of iron availability on microcystin production has received considerable attention following the early paper by Lukac and Aegerter [109], whose results first suggested an iron-chelating role of microcystin. Since this preliminary study, other research has further examined this relationship, such as Alexova et al. [68] and Yeung et al. [107]. Other trace metals have received much less attention, or in some cases none.

Culture-based experiments form most of the literature on cyanobacteria–metal interactions (≈63% of the studies from Table 1). While culture experiments often demonstrate unambiguous relationships between a single species growth and a given micronutrient, as demonstrated by Fujii et al. [70], it is also important to examine these relationships under field conditions which take into account environmentally relevant concentrations of trace metals, particularly as selective pressures and behaviours of culture-raised organisms can differ from those in natural systems [94]. Nutrient amendment bioassays are a useful tool in bridging the gap between culture and field studies, and have been used effectively in studies such as de Wever et al. [64] and Zhang et al. [37]. However, Nogueira et al. [150] outlines how the incubation time, sample volume and pre-filtration process of small-scale mesocosms may alter how representative the system is of the original community. 

It is unclear how regularly cyanobacterial blooms are limited by trace metals in natural systems. Field monitoring studies examining trace metal fluxes and cyanobacterial bloom dynamics, such as in Baptista et al. [7], are an important missing piece in the literature and must be extended to include a greater variety of systems and locations to link and validate the results of culture and bioassay studies. This is particularly important within the context of climate change, where higher temperatures, increased thermal stratification and flood-driven nutrient pulses are likely to intensify cyanobacterial blooms [22]. We also require a better understanding of the quantity of trace metals required to support cellular functions of cyanobacteria. This information would allow the development of a model that predicts scenarios where trace metals may become limiting. These gaps in the literature demonstrate a need for further study to fully understand how cyanobacteria and their toxins are influenced by trace metals.

## Figures and Tables

**Table 1:** Summary of published literature that assessed the effect of trace metals on the growth and toxin production of freshwater cyanobacteria. Y, limitation was observed for this element; C, colimitation with N and/or P was observed; N, no limitation was observed; T^+^, addition of element had a positive effect on cyanotoxin production; T^−^, limitation of nutrient increased toxin production; T±, no effect.

Location	Taxa	Co	Cu	Fe	Mn	Mo	Zn	Mix	Study
Culture	*Microcystis aeruginosa*			T^−^					Alexova et al. [68] (2011)
Culture	*Microcystis aeruginosa*			T^+^					Amé and Wunderlin [132] (2005)
Culture	*Anabaena* spp.					C			Attridge and Rowell [133] (1997)
Canadian Shield lakes	Pico-cyanobacteria			C					Auclair [134] (1995)
Torrão reservoir	*Microcystis aeruginosa*	N	N	N	N		N	N	Baptista et al. [7] (2014)
Culture	*Anacystis* sp.				Y				Cheniae and Martin [135] (1967)
Lake Tanganyika, East Africa	Pico-cyanobacteria			Y, C					de Wever et al. [64] (2008)
Lake Waihola, New Zealand	*Anabaena flos-aquae*	Y	Y	N	Y			Y	Downs et al. [35] (2008)
Lake Mahinerangi, New Zealand		N	N	N	N			N	
Culture	*Microcystis aeruginosa*			Y					Fujii et al. [70] (2016)
Culture	*Nostoc* sp.					C			Glass et al. [71] (2010)
Culture	*Microcystis aeruginosa*		T±				T±		Gouvêa et al. [108] (2008)
Culture	*Phormidium autumnale*		Y, T±	Y, T±					Harland et al. [72] (2013)
Lake Erken, Sweden	*Gloeotrichia echinulate*			C					Hyenstrand et al. [136] (2001)
Lake Erken, Sweden	*Gloeotrichia echinulate*			C				N	Karlsson-Elfgren et al. [137] (2005)
Culture	*Microcystis novacekii*			Y, T^+^					Li et al. [78] (2009)
Culture	*Microcystis aeruginosa*		N, T±	Y, T^−^	N, T±		Y, T±		Lukac and Aegerter [109] (1993)
Lake 227, Experimental Lakes Area	*Aphanizomenon schindlerii*			Y					Molot et al. [81] (2010)
	*Anabaena flos-aquae, Synechococcus*			Y					
Culture	*Anacystis nidulans*			Y		N			Peschek [138] (1979)
Culture	*Microcystis aeruginosa*		N				Y, T^+^		Polyak et al. [86] (2013)
Culture	*Synechocystis*				Y				Salomon and Keren [105] (2011)
Culture	*Microcystis aeruginosa*			T^−^					Sevilla et al. [82] (2008)
Laurentian Great Lakes	Total cyanophyta			C					Sorichetti et al. [139] (2014)
Culture	*Anabaena oscillarioides*					C			ter Steeg et al. [91] (1986)
Culture	*Microcystis aeruginosa*			T^+^					Utkilen and Gjolme [106] (1995)
Clear Lake, California	*Aphanizomenon flos-aquae*			C					Wurtsbaugh and Horne [140] (1983)
Culture	*Microcystis aeruginosa*			Y, T^−^					Yeung et al. [107] (2016)
Lake Taihu, China	Total cyanophyta	N	Y, C	Y, C	N	N			Zhang et al. [37] (2019)
	*Microcystis aeruginosa*	N	Y, C	C	N	N			
